# Multimodal Feature Fusion Method for Unbalanced Sample Data in Social Network Public Opinion

**DOI:** 10.3390/s22155528

**Published:** 2022-07-25

**Authors:** Jian Zhao, Wenhua Dong, Lijuan Shi, Wenqian Qiang, Zhejun Kuang, Dawei Xu, Tianbo An

**Affiliations:** 1 School of Cyber Security, Changchun University, Changchun 130022, China; zhaojian@ccu.edu.cn (J.Z.); daniel_daren@outlook.com (W.D.); qiangayo@163.com (W.Q.); xudw@ccu.edu.cn (D.X.); tambo@163.com (T.A.); 2 School of Computer Science and Technology, Changchun University, Changchun 130022, China; 3 Jilin Provincial Key Laboratory of Human Health Status Identification and Function Enhancement, Changchun 130022, China; shilj@ccu.edu.cn; 4 School of Electronic Information Engineering, Changchun University, Changchun 130022, China

**Keywords:** multi-modality, fine-grained, model fusion, emotion features, feature generation

## Abstract

With the wide application of social media, public opinion analysis in social networks has been unable to be met through text alone because the existing public opinion information includes data information of various modalities, such as voice, text, and facial expressions. Therefore multi-modal emotion analysis is the current focus of public opinion analysis. In addition, multi-modal emotion recognition of speech is an important factor restricting the multi-modal emotion analysis. In this paper, the emotion feature retrieval method for speech is firstly explored and the processing method of sample disequilibrium data is then analyzed. By comparing and studying the different feature fusion methods of text and speech, respectively, the multi-modal feature fusion method for sample disequilibrium data is proposed to realize multi-modal emotion recognition. Experiments are performed using two publicly available datasets (IEMOCAP and MELD), which shows that processing multi-modality data through this method can obtain good fine-grained emotion recognition results, laying a foundation for subsequent social public opinion analysis.

## 1. Introduction

Online public opinion gathers public views on public social events and has a huge impact on associated objects [1], such as the melamine event in 2008 [2], the Wei Zexi event in 2016 [3], and “COVID-19” in 2020 [4]. The exposure of these events has a heavy blow on relevant objects, revealing many problems existing behind the event and causing a significant impact on people’s life safety. People’s emotions have undergone great changes with the development and spread of public opinion, which has caused much trouble for public opinion management, including the World Health Organization and governments of different countries [5]. In addition, online public opinion has caused great distress for people to analyze online public opinion because of its attributes of complex content, the coexistence of truth and falsehood, and easy dissemination [6]. As an important part of public opinion analysis, emotion recognition plays an important role in the field of artificial intelligence, which still remains a challenging task even with the development of deep learning and natural language processing. The main reason is that there are many ways and characteristics of expressing emotion, such as implicit emotion, dialogue emotion, and so on. We can capture emotional features by studying different ways, such as speech features, video features, facial features, EEG features [6,7,8,9], etc. Therefore, fine-grained multi-modal emotion recognition has become one of the current hotspots in the field of emotion analysis. Among them, for the selection of multi-modal data, speech is the easiest to obtain, and it is also the most widely used mode in people’s daily communication and there are also a large number of emotional features in the speech signal, so emotion analysis is the core and hotspot in the field of multi-modal research [10,11,12].

In the field of multi-modal emotion analysis, there are many research models for different modalities. Wollmer et al. [13] fused audio and video modalities for the first time and used bidirectional long and short-term memory neural networks to conduct multi-modal emotion analysis, whose experimental results showed that this multi-modality recognition effect was superior to the single-modality results. Morency et al. [14] conducted validation experiments for multi-modality, and the results demonstrated that a joint model integrating video, audio, and text features could be effectively used to identify emotions in online videos. Afterward, Poria et al. [15] proposed a new approach for multi-modal emotion analysis, which consisted of collecting emotion from web videos by presenting a model that used audio, video and text modalities as information sources. Soleymani et al. [16] proposed a multi-modal emotion data analysis framework to retrieve user opinions and sentiments from video content. Poria et al. [17] proposed a multi-modality analysis model based on LSTM that enables utterances to capture contextual information from their surroundings in the same video, thereby aiding emotion analysis. In 2018, Majumder et al. [18] proposed a novel feature fusion strategy, which is performed hierarchically by first fusing the two modes together and then fusing the third pattern to provide a new direction for multi-modal feature fusion. More and more people study different model fusion methods and optimize them continuously, but the problem of sample imbalance in the data set itself, as well as the emotion feature retrieval method that restricts a single modality in the process of modal fusion, are not analyzed.

Based on this problem, the main contributions of this paper are as follows:

(1) In the text and speech models, the paper first analyzes the speech emotion feature retrieval method, which restricts the modal fusion in the existing modal fusion method, analyzes the emotion features in the speech signal, and then puts forward the MA2PE speech feature retrieval method.

(2) As for the problem of sample disequilibrium, several common data processing methods for sample disequilibrium are analyzed, and SOM oversampling method are finally proposed.

(3) A fine-grained emotion recognition method for both text and speech modalities of sample disequilibrium data is proposed and validated on IEMOCAP and MELD datasets, proving that this method outperforms the existing models.

## 2. Data Preprocessing and Model Architecture

### 2.1. Audio Feature Extraction Method–MA2PE

The traditional audio feature retrieval method MFCC obtains audio features with loss in treble or bass more or less. Meanwhile, the length of the generated audio features is also different due to the different lengths of each audio segment in the dataset; therefore, its length needs to be processed in a cutting or filling manner before the retrieved audio features are sent into the model for analysis, which to a large extent makes audio features have different deletions or redundancies [19,20,21].

Based on this, a new feature retrieval method, MA2PE, is proposed, which adopts a series of operations to retrieve and convert all speech data of different lengths into 8-dimensional feature vectors. The specific operation methods are as follows:

First, a 44.1 kHz sampling rate is used to read the time series of each audio, and the mean and standard deviation of the absolute value of each audio time series are obtained as two of the features, which not only retain other features but also play a role in integrating all length vectors, and the mean and standard deviation can also reflect certain feature differences.

Second, because the energy of the speech signal is related to its tone, thus it can be used to detect certain elevated emotions, such as anger, excitement, etc., as the tone and loudness of voices emitted by people in the case of anger will be different. Therefore, the following formula is used to calculate the root mean square of each audio spectral feature frame by frame and calculate the corresponding standard deviation to learn and represent the tone and loudness features of speech signals.

(1) $$E=\sqrt{\frac{1}{n}\sum_{i=1}^{n}y{\left[i\right]}^{2}}$$

Here *n* denotes the length of the time series, and y[i] denotes the input signal.

Then, we obtained the ratio of mute state to the total state in the audio signal as the fifth feature because of the fact that our speech rate will also vary in different emotional states, which leads to different proportions of mute state and it is significant for us to learn emotion features. Besides, we also calculate the harmonic energy in the time-frequency signal as the sixth feature. When people change their emotions, there will be changes in harmonics, which provides a reference for us to do fine-grained emotion analysis.

Finally: we all know that the waveform generated by articulation changes with our mood, which is the treble signal. There are many commonly used treble detection algorithms, including the modified autocorrelation function method (MACF) and normalized cross-correlation function method (NCCF), for example. This paper adopts the autocorrelation algorithm based on the center clipping frame, which is calculated by the following formula:

(2) $$res\left[n\right]=\left\{\begin{array}{c} y\left[n\right]-C_{l},y\left[n\right]\geq C_{l}\\ 0,|y\left[n\right]|<C_{l}\\ y\left[n\right]+C_{l},y\left[n\right]\leq C_{l} \end{array}\right.$$

where y[n] is the input signal and $C_{l}$ is half of the mean value of the input signal. We also put the calculated autocorrelation coefficient res[n] and the normalized value into the features of speech emotion.

Through the above methods, the 8-dimensional feature retrieval method of MA2PE can be obtained.

### 2.2. Oversampling Processing Method Based on SOM

The traditional sample disequilibrium processing method includes an oversampling method, undersampling method, and reweighting method [22,23,24], among which the traditional oversampling and undersampling method is to copy and delete the original sample. However, this method of obtaining text features causes repetition and waste of data features and cannot play a great role in the learning of fine-grained emotion features, while the multi-modal reweighting method also has problems more or less [25,26,27]. Therefore, this paper proposes a SOM oversampling method - an oversampling method based on TF-IDF synonymous substitution. It uses the commonly used text segmentation method to segment the text, obtains different keywords through TF-IDF, expands data according to the needs of the text, finds multiple alternative topics from the big data thesaurus, and replaces them to generate new sample data. In addition, through the text generated according to the sample and the voice data corresponding to the text of the original data, the package in the moviepy library is used to modify the audio content without changing audio information such as pitch, pitch, etc. The characteristics of the new samples generated by this method are different from those generated by the previous method, and they are diverse. In this way, if they are sent to the model for learning, more characteristics can be obtained, which is very useful for the analysis of fine-grained emotion features.

### 2.3. Modal Fusion Method

**Figure 1:**
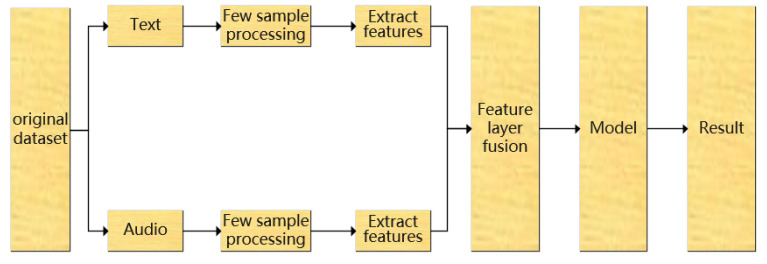
Feature Layer Fusion Scheme for Fewer Samples.

**Figure 2:**
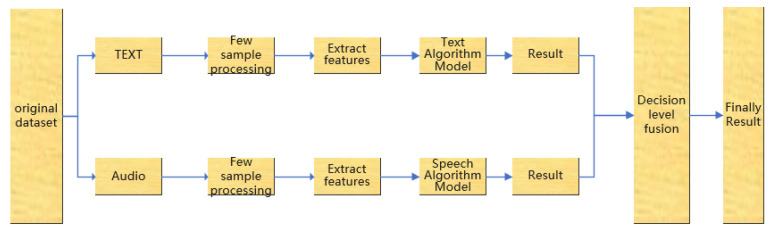
Decision Level Fusion Approach for Few Shot.

For the modal fusion method, the fusion methods based on the decision layer and the feature layer are selected respectively for the analysis and verification of the comparative experiments. The following two fusion models are designed respectively, as shown in Figure 1 and Figure 2.

Among the fusion methods based on the decision layer, there are several common combination methods, including the voting method, fuzzy integration method and D-S evidence reasoning method. However, this classification method cannot allocate appropriate weights based on the features of each modality sufficiently [28]. Hence, this paper proposes a dynamic weight allocation method. According to the classification results of speech and text, the same weight is given first, and the weight ratio is adjusted continuously by comparing the correct results with the linear weighting plan, and finally, the suitable weight is obtained.

### 2.4. Model Framework

**Figure 3:**
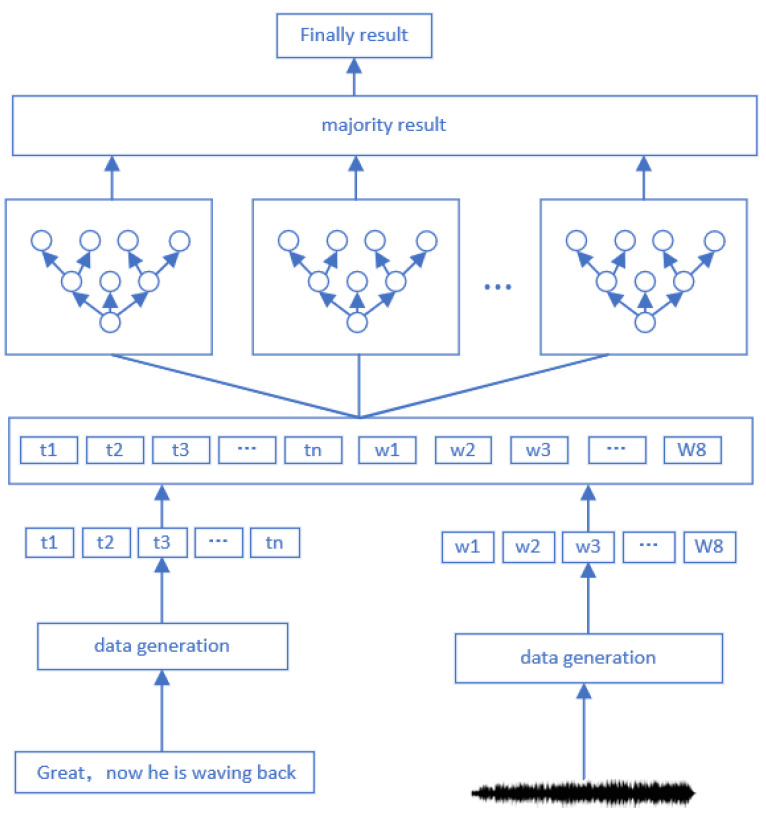
Multi-modal fine-grained emotion classification model based on sample disequilibrium.

The multi-modal fine-grained emotion analysis model based on feature layer fusion designed in this paper is mainly composed of four modules: processing module of few shot data, text feature retrieval module, speech feature retrieval mode, and multi-modal feature layer fusion module. As shown in Figure 3, the model processes text and speech modalities separately.

According to the text modality, firstly, the text data expansion method is used to amplify the few shot data in the sample disequilibrium data, and then the amplified text is feature retrieved.

For the speech modality, the corresponding audio data generation method is used to generate the corresponding text, and then the audio data feature is retrieved. Because the audio feature dimension proposed in this paper is only 8-dimensional, it is easy to complicate the features by selecting dot multiplication or dot product operation. Thus, finally, the feature layer is fused by simple stitching and then sent into the model for analysis to obtain the required results.

## 3. Parameter Setting and Result Analysis

### 3.1. Datasets

Due to the lack of emotional data of existing network discourse, We mainly use two multi-modal dialogue emotional data sets, MELD and IEMOCAP, for experiments. The MELD dataset comes from about 13,000 utterances from 1433 dialogues from the TV series Friends. It is divided into seven categories in total, as shown in Table 1. The IEMOCAP dataset contains about 12 h of audio-visual data, including video, voice, facial motion capture, and text content. Contains ang, happy, sad, fear, surprise, and neutral six emotions; this study focuses on two modes, the corresponding amount of data (text and audio) shown in Table 1.

### 3.2. Experiment Procedures

**Figure 4:**
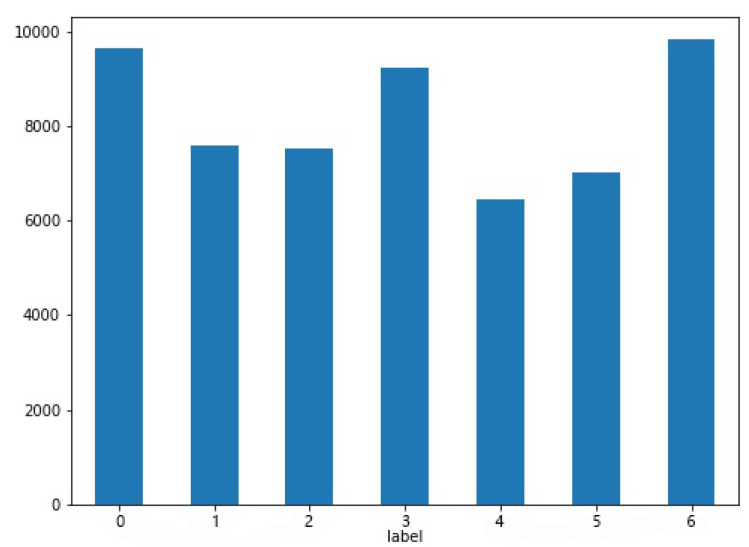
The representation of sample data volume after over-sampling in MELD dataset (where 0–6 in label represents six emotional labels: ‘anger’, ‘disgust’, ‘fear’, ‘joy’, ‘neutral’, ‘sadness’, ‘surprise’).

(1) For the few shot data, the oversampling method is used to generate text and speech data corresponding to the results, respectively. The specific generation results are shown in Figure 4.

(2) Text dialogues are divided into tokens and each word becomes lowercase.

(3) As for the text processing scheme, TF-IDF is used to obtain the weight matrix of the text.

(4) Set the audio sampling rate to 44,100 Hz.

(5) Audio features were retrieved by using the audio feature retrieval method of MA2PE.

(6) A new two-modality feature is generated by simply stitching the generated speech and text features.

(7) The model is trained by using the algorithm of Random Forest and tested on the test set, and the results of each modality are calculated.

### 3.3. Experiment Results and Analysis

In this part, the results of previous experiments are presented and analyzed. Table 2 and Table 3 present the results of the MELD data set and IEMOCAP data set in the case of text, speech, and multi-modal fusion, respectively. Because there are differences in the data volume between MELD and IEMOCAP, and there are differences in the sample disequilibrium proportion, the MELD dataset and IEMOCAP dataset will be analyzed separately in this paper.

#### 3.3.1. Experiment Results and Analysis of MELD Data Set

**Figure 5:**
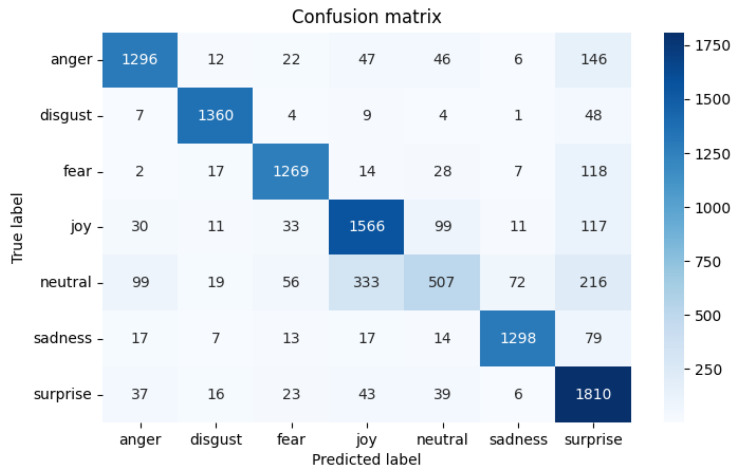
Results Heat Map of MELD Data Set Fusion Model.

This paper first conducts an evaluation experiment of model comparison on the MELD data set and the experimental results are shown in Table 2. The heat map of the results on the multi-modal fine-grained emotion analysis structure based on feature layer fusion is shown in Figure 5. The benchmark models of this experiment are the Text-CNN, HiGRU-sf [29], cMKL, bcLSTM [17], and DialogueRNN [15] models shown in Table 2, all of which are the optimal results of multi-modal fine-grained analysis of the MELD data set. Other models, including SVC, LR, etc., are all models used after over-sampling the samples. According to the results in the table and the following analysis results, we can draw the following conclusions:

**Table 2:** MELD dataset analysis result table.

Methods	Model	Anger	Disgust	Fear	Joy	Neutral	Sadness	Surprise	acc	w_avg
Text-CNN	text	34.49	8.22	3.74	49.39	74.88	21.05	45.45	—	55.02
HiGRU-sf	text	21.16	0	2	47.01	91.56	0.48	40.93	—	58.58
cMKL	Text + audio	39.50	16.10	3.75	51.39	72.73	23.95	46.25	—	55.51
bcLSTM	text	42.06	21.69	7.75	54.31	71.63	26.92	48.15	—	56.44
	Audio	25.85	6.06	2.90	15.74	61.86	14.71	19.34	—	39.08
	text + audio	43.39	23.66	9.38	54.48	76.67	24.34	51.04	—	59.25
DialogueRNN	text	40.59	2.04	8.93	50.27	75.75	24.19	49.38	—	57.03
	Audio	35.18	5.13	5.56	13.17	65.57	14.01	20.47	—	41.75
	text + audio	43.65	7.89	11.68	54.40	77.44	34.59	52.51	—	60.25
LR	audio	21.47	0.00	0.00	20.71	27.54	27.79	19.67	21.05	17.11
	text	84.71	93.71	88.96	74.28	60.21	90.50	67.69	80.13	79.76
	text + audio	79.14	93.14	88.01	73.96	62.41	90.20	71.74	79.94	79.39
	text + audio (back)	87.76	93.75	89.24	77.65	70.71	92.98	63.37	82.32	82.60
MLP	audio	23.64	25.69	25.78	21.32	22.22	27.08	23.52	24.07	24.04
	text	89.68	96.03	92.81	79.95	84.91	93.97	68.14	84.4	85.69
	text + audio	95.10	95.95	97.94	86.02	82.40	94.23	81.80	90.23	90.24
	text + audio (back)	93.08	95.70	93.22	86.64	93.33	96.88	70.23	87.51	88.94
MNB	audio	20.03	0	0	1	0	0	17.2	17.86	22.73
	text	81.40	90.69	82.08	64.91	60.77	91.19	63.68	75.62	75.85
	text + audio	84.00	93.10	82.33	61.22	63.97	93.46	63.31	75.34	76.56
	text + audio (back)	83.32	92.89	83.49	65.56	76.16	93.82	65.12	77.47	79.02
SVC	audio	20.80	46.43	28.39	21.30	0.00	29.16	21.10	21.71	23.79
	text	90.24	95.32	88.96	75.48	67.71	93.17	70.24	82.59	82.71
	text + audio	89.87	94.31	89.37	77.18	68.79	92.65	71.43	83.02	83.07
Our model	audio	84.91	97.95	97.91	82.12	87.96	90.69	86.42	88.88	89.08
	text	87.74	94.74	90.93	80.01	77.46	93.44	71.46	84.08	84.50
	text + audio	93.80	99.44	99.32	86.14	96.77	97.88	89.83	93.73	94.13

**Figure 6:**
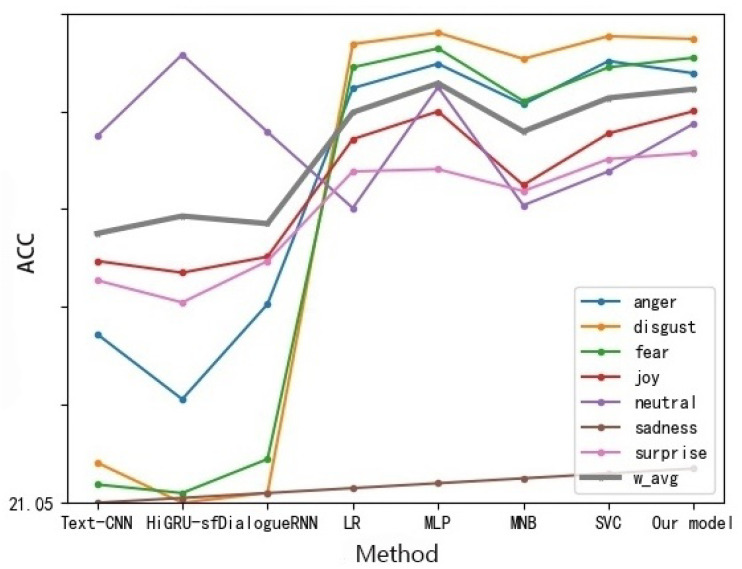
Fine-grained Emotion Accuracy Rate of Different Methods of MELD Data Set (Text).

As for text data: we can observe that the accuracy rate of almost all emotions is higher than that of audio, which may be due to the fact that the features obtained from text are richer than those of audio. As shown in Figure 6, compared with the benchmark model, we can find that almost all oversampling processing methods are superior to other methods, while the multi-modal fine-grained emotion analysis method based on feature layer fusion proposed in this paper can bring better results than other methods, and the classification effect is also the best. The accuracy rate of all emotion categories is about 80%, whether acc or w_avg is used as the evaluation index. It is the best category compared with other methods because the classification effect reaches 84.50%.

**Figure 7:**
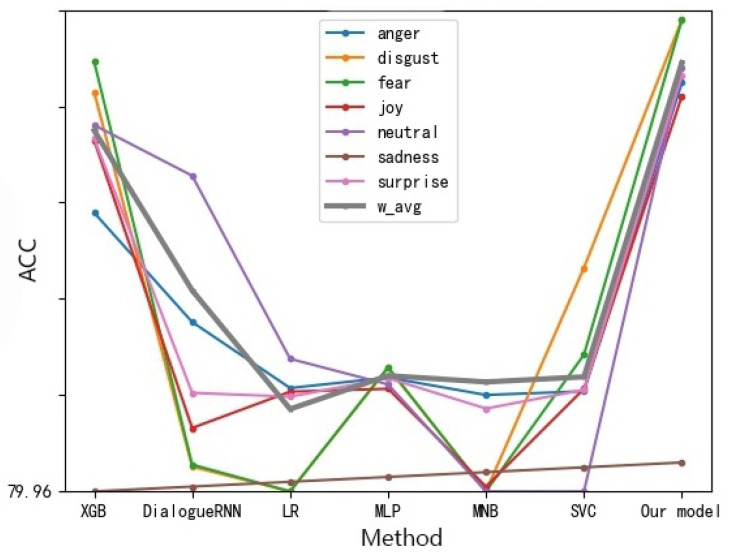
Fine-grained Emotion Accuracy Rate of Different Methods of MELD Data Set (speech).

As for audio data: Compared with the original audio feature retrieval method, the current one is more general and with a better result, especially for the problem of sample disequilibrium, which can effectively prevent the occurrence of the overfitting phenomenon. As shown in Figure 7, we can observe that the results of logistic regression, multi-layer perceptron and support vector machines are relatively low, while the results of ours are the highest, whose overall classification effect reaches 88.88%, and its accuracy rate of aversive emotion reaches 97.95% at the highest. In addition, it is also found that this method has high accuracy in judging negative emotions. Because of the characteristics of audio signals, it is not easy to learn but very easy to overfit. The commonly used speech feature retrieval method, such as MFCC, retrieves features in various lengths due to the problem of audio length. For the unity of the model, we need to cut or fill, which leads to redundancy or loss of feature information. While our feature retrieval method can effectively solve this problem and also retain the audio features. Therefore, it can be found that the accuracy rate of audio features retrieved on MA2PE is higher than that of text.

**Figure 8:**
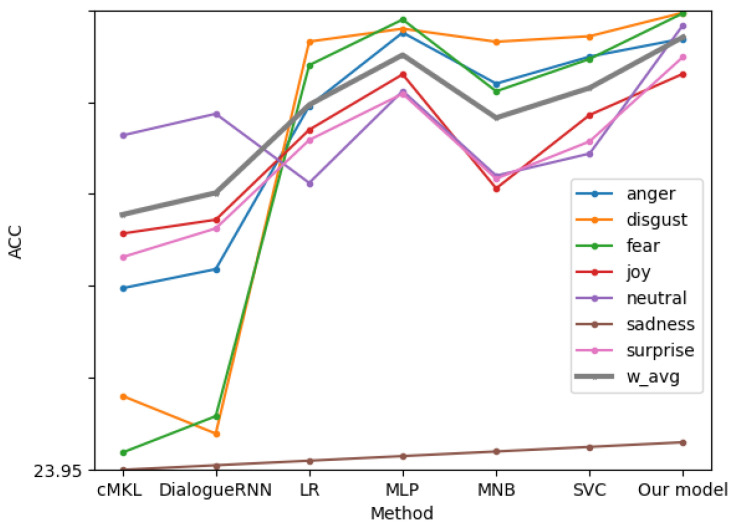
Fine-grained Emotion Accuracy Rate of Different Methods of MELD Data Set (Multimodalities).

For multi-modal data sets: we can find the results of modal fusion, whether feature layer fusion or decision layer fusion is superior to a single modality. In the figure, text + audio (back) refers to decision layer fusion, while text + audio refers to feature layer fusion method. Besides, the best method improves the accuracy rate by almost 10%. Compared with the weighted fusion method based on the decision layer, the method of feature layer fusion is similar in effect, but the method of decision layer fusion requires reasonable learning of the weights of different modalities, which is very time-consuming. While the feature layer fusion method of stitching not only retains the features of the two modalities of text and speech but also does not cause great time consumption of the fused features because the speech features are only 8-dimensional. Therefore, it is a more appropriate choice. Compared with other basic models, our model has better results in any kind of fine-grained emotional data set, and the final classification effect reaches 94.13%, which is 33.88% higher than the best DialogueRNN model of the benchmark model. From Figure 8, we can also find that the overall results of all the models processed by the oversampling method are superior to those of the benchmark model.

#### 3.3.2. Experiment Results and Analysis of IEMOCAP Data Set

**Figure 9:**
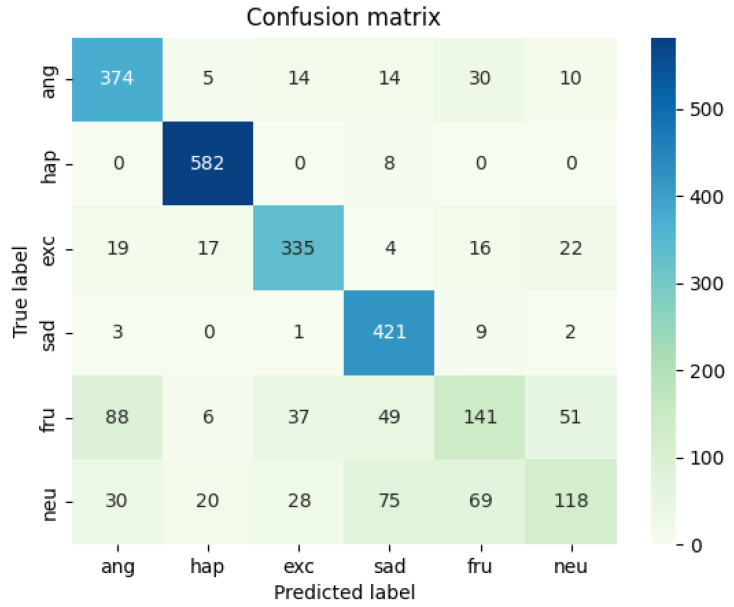
Result Heat Map of Fusion Model of IEMOCAP Data Set (out model).

**Figure 10:**
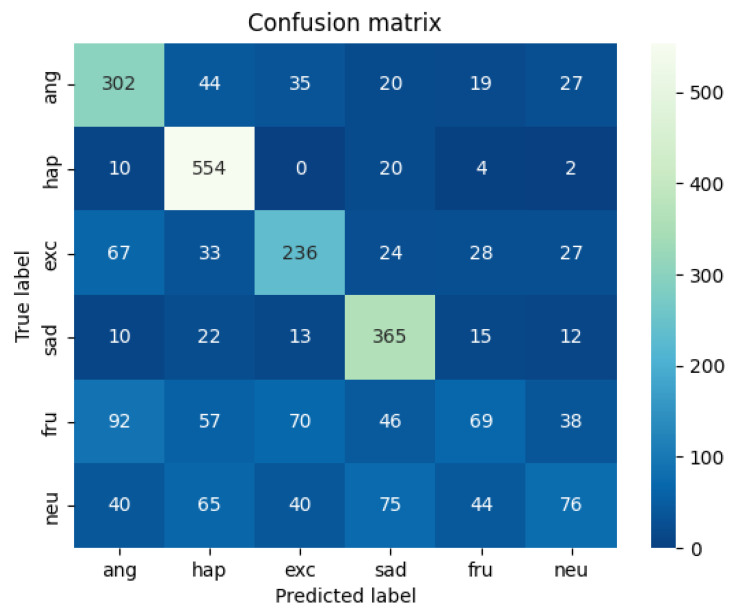
Speech Feature Heat Map of IEMOCAP Data Set (our model).

A comparison and evaluation experiment of the model is carried out on the IEMOCAP data set and the experimental results are shown in Table 3. The heat map of the results on the multi-modal fine-grained emotion analysis structure based on feature layer fusion is shown in Figure 9 and Figure 10. As shown in Table 3, the benchmark models of this experiment are HiGRU [29], HiGRU-sf [29], mement [30], cLSTM [17], TFN [31], MFN [32], CMU [33], and ICON [10], all of which are the optimal results for the multi-modal fine-grained analysis of the IEMOCAP data set. Other models, including SVC, LR, etc., are all models used after over-sampling the samples. According to the results in the table and the following analysis results, we can reach the following conclusions:

**Table 3:** IEMOCAP dataset analysis result table.

Method	Model	ang	hap	exc	sad	fru	neu	acc	w_avg
HiGRU	text	64.12	39.86	62.21	78.37	60.37	62.50	—	62.52
HiGRU-sf	text	71.18	51.75	62.88	70.20	61.68	64.84	—	64.06
LR	audio	40.07	24.36	16.67	42.85	15.38	0	32.44	24.47
	text	72.71	69.36	69.89	61.78	49.03	45.37	64.01	62.94
	text + audio	73.18	73.66	73.35	68.77	51.87	51.65	67.82	66.71
MLP	audio	40.22	25.91	28.86	42.20	17.41	46.66	34.10	33.07
	text	80.32	88.61	77.19	60.53	55.37	50.60	71.43	71.37
	text + audio	77.66	89.64	84.92	82.12	58.41	54.33	77.94	76.48
MNB	audio	69.76	23.24	0	0	0	0	24.78	17.28
	text	73.58	64.17	65.35	57.35	49.64	46.24	61.47	60.59
	text + audio	70.97	60.27	69.16	67.54	50.64	55.87	63.05	62.79
SVC	audio	39.66	24.73	100	39.37	11.36	0	32.67	36.57
	text	75.29	75.33	69.02	68.60	50.00	46.69	67.62	66.08
	text + audio	73.22	83.18	77.02	68.89	50.85	49.61	70.51	69.07
XGB	audio	59.81	62.93	55.34	67.40	29.60	39.72	58.96	54.13
	text	70.16	42.78	71.30	59.61	47.66	45.80	51.46	55.91
	text + audio	55.20	58.89	73.67	63.31	47.39	66.40	60.16	60.68
Menment	Text + audio + video	67.1	24.4	65.2	60.4	68.4	56.8	—	59.9
	Text + audio + video	70.0	25.5	58.8	58.6	67.4	56.5	—	59.8
	Text + audio + video	69.1	23.2	63.1	58.0	65.5	56.6	—	58.8
	Text + audio + video	72.3	24.0	64.3	65.6	67.9	55.5	—	60.1
CMU	Text + audio + video	67.6	25.7	69.9	66.5	71.7	53.9	—	61.9
ICON	Text + audio + video	68.2	23.6	72.2	70.6	71.9	59.9	—	64.0
Our model	audio	57.97	71.67	59.89	66.30	38.55	41.76	61.62	57.88
	text	77.46	84.78	77.35	55.81	56.45	53.72	69.89	69.77
	text + audio	72.76	92.38	80.72	73.73	53.21	58.13	75.87	73.93

**Figure 11:**
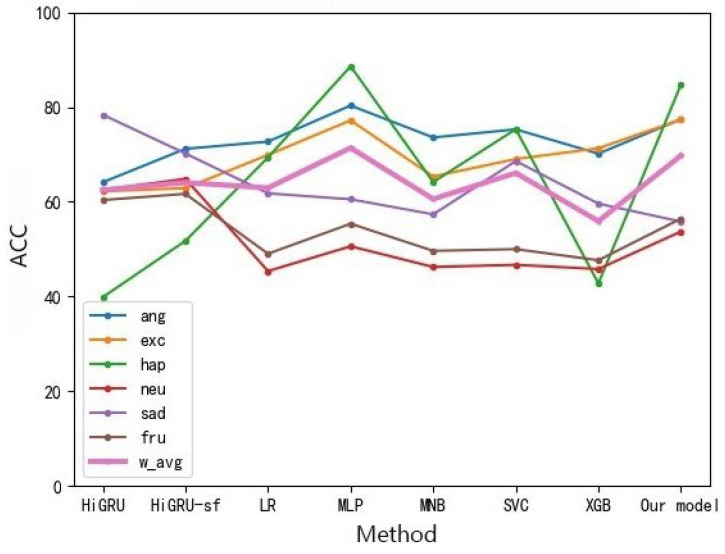
Fine-grained Emotion Accuracy Rate Analysis of Different IEMOCAP Methods (Text).

Analysis of the text: As shown in Figure 11, the method of oversampling and the method without oversampling are compared, and the results of the oversampling method are greatly improved. Compared with other models of oversampling, the emotion analysis model based on feature layer fusion proposed in this paper can achieve the classification effect at 67.77 due to the vast majority of models.

**Figure 12:**
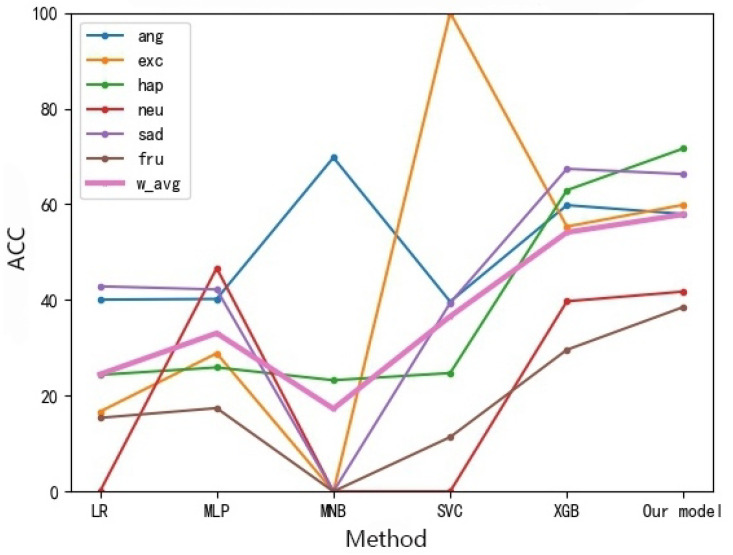
Fine-grained Emotion Accuracy Rate Analysis of Different MOCAP Methods (Audio).

Analysis of the audio: Because there is no benchmark model for comparison, we compare different models of the same processing method. As shown in Figure 12, the SVC of the emotion analysis model based on feature layer fusion proposed in this paper is higher than that of other models. Relatively speaking, our model is superior to other models because of its better stability.

**Figure 13:**
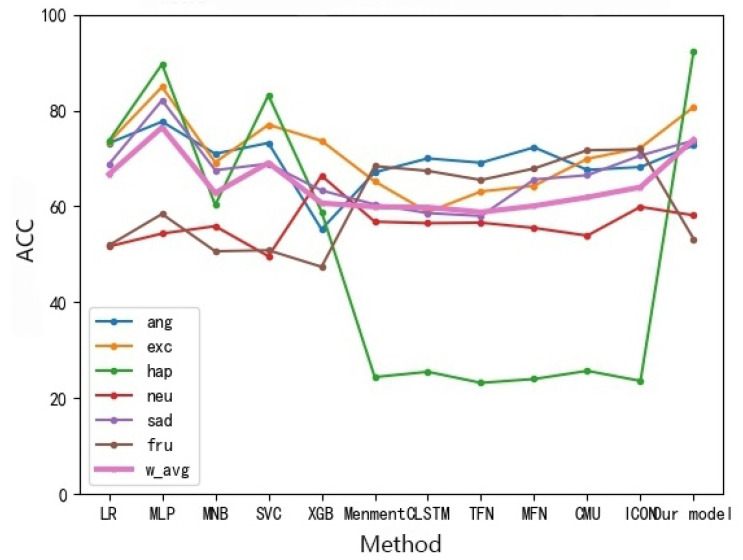
Fine-grained Emotion Accuracy Rate Analysis of Different MOCAP Methods (Multimodalities).

Analysis of multi-modal fusion: As shown in Figure 13, compared with the other three modality fusion benchmark models, the fusion results of our two modalities are superior to the fusion results of the three modalities, showing good results in various emotional result features. Compared with other models in the same processing, the classification effect of our model is basically the same as other models.

However, by comparing the classification effect of IEMOCAP and MELD data, we find that this method has a great effect on the MELD data set and a good effect on the IEMOCAP data set. Relatively speaking, its classification effect is poor. Therefore, we analyze the differences between the two data sets. By comparing the results of the two data sets shown in Table 1, we observe that the MELD data set is very wide and with stronger data disequilibrium performance. After data expansion, the data volume is basically around 8000, as shown in Figure 4, while the data disequilibrium of IEMOCAP data itself is not obvious. After data expansion, the data volume basically remains around 1500. Compared with the MELD data set, the data volume is much less and the samples are of relative equilibrium, so it can be concluded that the multi-modal fine-grained emotion analysis model based on feature layer fusion proposed in this paper has a better effect on the sample disequilibrium data.

## 4. Conclusions

This paper mainly aims at the limitations of traditional speech emotion features existing in multi-modal emotion recognition and the problem of accuracy rate decline caused by disequilibrium sample data of multi-modal research. Besides, it further studies speech emotion features and sample disequilibrium problems before a speech emotion feature retrieval method of MA2PE and an oversampling method of SOM are respectively proposed. The MA2PE method can express emotion features better, which can improve the accuracy rate of emotion expression by nearly 30%. SOM oversampling method is used to amplify samples and improve the utilization rate of data. In addition, it is verified in two public data sets—MELD and IEMOCAP, whose results show that it can achieve good results.

Based on the methods of MA2PE and SOM, we propose a multi-modal fine-grained emotion analysis model based on feature layer fusion. By combining the first two methods, the model fuses text features and speech features at the feature layer and puts them into the model for analysis. Through the analysis results of different methods of the two data sets, we can conclude that this method has a good effect on the study of multi-modal data based on sample disequilibrium, and the more unequal the data are, the better the effect is.

This paper mainly analyzes public emergencies in voice and text modes, but public opinion events also include other modalities, such as video, expression, etc., which also have important guidance for the event results. Therefore, in the subsequent research, more attention will be paid to the study of public opinion data of more modalities.

## Figures and Tables

**Table 1:** IEMOCAP dataset and MELD dataset data volume.

Emotion Category	IEMOCAP Total Data Volume	MELD Total Data Volume
ang	1103	1607
Hap/joy	595	2308
Sad/sadness	1041	1002
fear	1084	358
surprise	1849	1636
neutral	1708	6436
disgust	—	361

## Data Availability

Not applicable.
